# Supplementary material for: Negative cooperativity between Gemin2 and RNA provides insights into RNA selection and the SMN complex's release in snRNP assembly
Source: Nucleic Acids Res. 2019 Dec 4;48(2):895–911. doi: 10.1093/nar/gkz1135 (PMC6954390; doi:10.1093/nar/gkz1135)
Supplement: gkz1135_Supplemental_File [file gkz1135_supplemental_file.pdf]

SUPPLEMENTARY MATERIALS

Negative Cooperativity between Gemin2 and RNA provides Insights into RNA Selection and the SMN Complex's Release in snRNP Assembly

Hongfei Yi[1], Li Mu[1], Congcong Shen, Xi Kong, Yingzhi Wang, Yan Hou and Rundong Zhang*

Containing

7 supplemental tables (Tables S1-7),

8 supplemental figures (Figures S1-8)

Table S1. Data Collection and Refinement Statistics

**Variable protein components***			
	Complex A	Complex B	Complex C
Gemin2	FL(1-280)	ΔN39 (40-280)	ΔN39 (40-280)
SmD1	ΔC(1-82)	ΔC(1-82)	ΔC(1-82)
SmG	Yes	No	Yes
**Data collection**			
Wavelength (Å)	0.97853	0.97853	0.97853
Space group	$P2_12_12_1$	$P2_12_12_1$	$P2_12_12_1$
Unit cell (Å):	83.32,	83.17,	82.96,
a, b, c	115.76,	114.09,	114.12,
	128.21	125.39	130.14
Highest resolution (Å)[$]:			
a*, b*, and c*	3.3, 3.5, 3.9	3.4, 3.4, 4.0	3.1, 3.2, 3.6
Unique reflections	15470	14569	18768
Completeness (%)	98.2 (94.6)[a]	99.5(97.9)[b]	97.8(95.2)[c]
	79.2(15.9)[d]	84.2(35.5)[e]	83.1(28.8)[f]
R_{meas}	0.154 (0.905)	0.233(0.914)	0.166(0.885)
R_{pim}	0.048 (0.261)	0.087(0.297)	0.056(0.243)
Mean I/σ	13.6 (2.2)	12.9(2.0)	10.4(2.0)
Redundancy	12.4 (12.3)	10.8 (8.7)	12.4(12.7)
**Refinement Statistics**			
Resolution range(Å)	48-3.3	47-3.4	47-3.1
R factor (%)	20.5	19.1	17.2
R_{free} factor (%)	26.5	24.5	24.0
Number of reflections	15470	14569	18768
Number of atoms	4793	4346	4890
Rmsd bond length (Å)	0.0112	0.0122	0.0129
Rmsd bond angles (°)	1.576	1.624	1.477
Ramachandran plot (%)[#]:			
Favored,	93.9,	91.6,	94.7,
additional allowed,	5.9,	7.5,	4.6,
disallowed	0.2	0.9	0.7
PDB code	5XJQ	5XJS	5XJR

* Complexes A-C all contain SMN(residues 26-62), SmD2, SmF and SmE.

[$] Ellipsoidal truncation was performed on each data set due to serious anisotropic diffraction.

[a-c] For resolution range of 48-3.9, 47-4.0 and 47-3.6 Å respectively. The number in the parenthesis corresponds to the highest-resolution shell of 4.04-3.90, 4.12-4.00 and 3.72-3.60 Å respectively.

[d-f] For resolution range of 48-3.3, 47-3.4 and 47-3.1 Å respectively. The number in the parenthesis corresponds to the highest-resolution shell of 3.4-3.3, 3.52-3.40 and 3.2-3.1 Å respectively.

[#] gained from Coot program.

Table S2. RNA sequences used in this study.

Name	RNA sequence
9nt	AAUUUUUGA
3’Sm	GGGAAUUGAAAACUUUUCCCAAUACCCCAAUUUUUGA
U4	GGGAAUUGAAAACUUUUCCCAAUACCCCGCCGUGACGACUUGCAAUAU<br>AGUCGGCACUGGCAAUUUUUGACAGUCUCUACGGAGACUG
U4ΔSm	GGGAAUUGAAAACUUUUCCCAAUACCCCGCCGUGACGACUUGCAAUAU<br>AGTCGGCACUGGCAACCCCCGACAGUCUCUACGGAGACUG
U4-3’ss	GGGAAUUGAAAACUUUUCCCAAUACCCCGCCGUGACGACUUGCAAUAU<br>AGUCGGCACUGGCAAUUUUUGACAGUCUCUACGCUCUGAC
U4-3’Δ	GGGAAUUGAAAACUUUUCCCAAUACCCCGCCGUGACGACUUGCAAUAU<br>AGUCGGCACUGGCAAUUUUUGAC
U4-5’ss	GGGAAUUGAAAACUUUUCCCAAUACCCCAAUUUUUGACAGUCUCUACG<br>GAGACUG
U4-5’Δ	GGCAAUUUUUGACAGUCUCUACGGAGACUG
U4-5’Δ-3’ss	GGCAAUUUUUGACAGUCUCUACGCUCUGAC
flU4	GGGCAGCUUUGCGCAGUGGCAGUAUCGUAGCCAAUGAGGUCUAUCCGA<br>GGCGCGAUUAUUGCUAAUUGAAAACUUUUCCCAAUACCCCGCCGUGAC<br>GACUUGCAAUAUAGUCGGCACUGGCAAUUUUUGACAGUCUCUACGGAG<br>ACUG
flU4ΔSm	GGGCAGCUUUGCGCAGUGGCAGUAUCGUAGCCAAUGAGGUCUAUCCGA<br>GGCGCGAUUAUUGCUAAUUGAAAACUUUUCCCAAUACCCCGCCGUGAC<br>GACUUGCAAUAUAGUCGGCACUGGCAACCCCCGACAGUCUCUACGGAG<br>ACUG
flU4-spacer	GGGCAGCUUUGCGCAGUGGCAGUAUCGUAGCCAAUGAGGUCUAUCCGA<br>GGCGCGAUUAUUGCUAAUUGAAAACUUUUCCCAAUACCCCGCCGUGAC<br>GACUUGCAAUAUAGUCGGCACU**CCG**AAUUUUUGACAGUCUCUACGGAG<br>ACUG
flU4-spacer-3’ss	GGGCAGCUUUGCGCAGUGGCAGUAUCGUAGCCAAUGAGGUCUAUCCGA<br>GGCGCGAUUAUUGCUAAUUGAAAACUUUUCCCAAUACCCCGCCGUGAC<br>GACUUGCAAUAUAGUCGGCACU**CCG**AAUUUUUGACAGUCUCUACGCUC<br>UGAC

Red, Sm site. Blue, ΔSm. Cyan, spacer.

Table S3. The components and amounts used for gel filtration chromatographic (GFC) assay.

Input components (RNA colored in blue)	Amount used (molar ratio in last brackets)
D1s/D2 + F/E/G (5Sm)	200μg + 200μg (1:1)
Gemin2ΔN39	200μg
D1s/D2	1mg
F/E/G	1mg
3'Sm	10μg
5Sm + 3'Sm	400μg + 100μg (1:1)
5Sm + 9nt	400μg + 120μg (about 1:5)
Gemin2ΔN39/SMN$_{Ge2BD}$ + D1s/D2 + F/E/G (7SΔN preparation)	0.6mg + 1mg + 1mg (1:2:2)
7SΔN + 3'Sm	160μg + 40μg (about 1:1.5)
7SΔN + U4	160μg + 60μg (1:1)
(7SΔN + D3/B) + U4	(160μg + 60μg) + 60μg (1:1.5:1)
7SΔN + U4ΔSm	160μg + 60μg (1:1)
7SΔN + U4-3'Δ	160μg + 50μg (1:1)
7SΔN + U4-5'Δ	160μg + 40μg (1:2)
U4-5'Δ	5μg
7SΔN + U4-3'ss	160μg + 60μg (1:1)
7SΔN + U4-5'ss	160μg + 40μg (1:1)
7SΔN + U4-5'Δ-3'ss	80μg + 40μg (1:4)
7SΔN + U4-5'Δ-3'ss + U4-5'Δ	80μg + 15μg + 15μg (1:1.5:1.5)
5Sm + U4-5'Δ	400μg + 40μg (2:1)
5Sm + U4-5'ss	400μg + 80μg (2:1)
flU4	10μg
7SΔN + flU4	120μg + 120μg (1:1.6)
7SΔN + flU4ΔSm	100μg + 100μg (1:1.6)
7SΔN + flU4-spacer	120μg + 120μg (1:1.6)
7SΔN + flU4-spacer-3'ss	80μg + 80μg (1:1.6)
(7SΔN + D3/B) + flU4	(120μg + 60μg) + 120μg（1:1.5:1.6）
(7SΔN + D3/B) + flU4-spacer-3'ss	(80μg + 40μg) + 80μg (1:2:1.6)
7SΔN + (flU4-spacer + U4-5'Δ-3'ss)	80μg + (80μg + 16μg) (1:1.6:1.6)
7SΔN + (flU4-spacer-3'ss + U4-5'Δ)	80μg + (80μg + 16μg) (1:1.6:1.6)
(7SΔN + D3/B) + (flU4-spacer-3'ss + U4-5'Δ)	(80μg + 40μg) + (80μg + 16μg) (1:2:1.6:1.6)
(7SΔN + D3/B) + (flU4 + U4-5'Δ)	(100μg + 50μg) + (100μg + 20μg) (1:2:1.6:1.6)

Table S4. The GFC elution positions of the RNAs, proteins, and their complexes studied.

Components	Elution position (ml)	+7SΔN: Elution position (ml)	+5Sm: Elution position (ml)	+7Sm: Elution position (ml)
9nt	~19.6	Not formed	14.37	Not tested
3'Sm	16.64	Not formed	13.51	Not tested
U4	14.75	13.31	Not tested	13.43
U4ΔSm	14.91	Not formed	Not tested	Not tested
U4-3'ss	14.48	13.11	Not tested	Not tested
U4-3'Δ	15.66	Not formed	Not tested	Not tested
U4-5'ss	15.67	13.65(RNA+5Sm)	13.50	Not tested
U4-5'Δ	17.48	14.29(RNA+5Sm)	14.27	Not tested
U4-5'Δ-3'ss	16.62	13.97	Not tested	Not tested
flU4	13.08	12.26	Not tested	12.31
flU4ΔSm	12.93	Not formed	Not tested	Not tested
flU4-spacer	12.97	12.24(RNA+5Sm)	Not tested	Not tested
flU4-spacer-3'ss	12.90	~12.11	Not tested	12.14
7SΔN	13.78	----------	----------	----------
7S	13.61	----------	----------	----------
D1s/D2	16.63	----------	----------	----------
F/E/G	14.29(dimer), 16.62(monomer)	----------	----------	----------
D1s/D2/F/E/G(5Sm)	15.06	----------	----------	----------
Gemin2ΔN39/SMN_{Ge2BD}	15.66	----------	----------	----------

5Sm indicates D1s/D2 and F/E/G. 7Sm indicates 5Sm and D3(1-75)/B(1-91).

Table S5. The molar extinction coefficients of the proteins, protein complexes and RNAs studied.

Sample	Length	MW(Da)	Molar Extinction Coefficient ($mol/L\cdot cm^{-1}$)	
			at 280 nm	at 260 nm
**Protein:**				
SmD1s(1-82)	82	9287.57	1280	640
SmD2	119	13583.36	7210	3605
SmF	86	9724.38	12210	6105
SmE	92	10802.89	10930	5465
SmG	77	8552.85	120	60
SmD3(1-75)	75	8465.17	4080	2040
SmB(1-92)	92	10508.92	1640	820
D1s/D2	201	22870.93	8490	4245
SmF/E/G	255	29080.12	23260	11630
D3/B	167	18974.09	5720	2860
5Sm	657	74821.98	31750	15875
7Sm	824	93796.07	37470	18735
Gemin2ΔN39	242	27347.49	36970	18485
SMN(26-62)	37	4042.12	7090	3545
Gemin2ΔN39/SMN(26-62)	279	31389.61	44060	22030
7SΔN	936	106211.59	75810	37905
**RNA:**				
9nt	9	3043	40909	81818
3'Sm	37	12017.5	168182	336364
U4	88	28363	400000	800000
U4-3'ss	88	28363	400000	800000
U4-3'Δ	71	22914.5	322727.5	645455
U4-5'ss	55	17786.5	250000	500000
U4-5'Δ	30	9774	136363.5	272727
flU4	148	47593	672727	1345454
flU4ΔSm	148	47593	672727	1345454
flU4-spacer	148	47593	672727	1345454
flU4-spacer-3'ss	148	47593	672727	1345454

Note: The molar extinction coefficient (MEC) at 280nm of protein is calculated by Vector NTI software (Thermo Fisher Scientific). The MEC at 260nm of each protein is estimated as the half of the MEC at 280nm. The MEC of protein complex (in red) is simply the sum of its components.

The MEC at 260nm of RNA (in blue) is calculated on the basis of 1 OD_{260} ssRNA=40μg/ml=0.11mM (in nucleotides) and its length. The equation is MEC (at 260nm) = 1000 X RNA length/0.11. The MEC at 280nm of RNA is estimated as the half of the MEC at 260nm.

Table S6. The calculated concentrations of RNAs and RNA-protein complexes from GFC for single RNA's assembly into Sm subcore and core.

the sample incubated with 7SΔN for GFC	OD(mAu) at 260nm			MEC [at 260nm]			Concentration(nM)		
	RNA-protein	RNA	7SΔN*	RNA-protein#	RNA	7SΔN	RNA-protein	RNA	7SΔN*
U4	24.3	85.1	9.6	837905	800000	37905	29	106	253
U4ΔSm	0.0	74.4	9.6	837905	800000	37905	0	93	253
U4-3'ss	47.9	68.4	10.5	837905	800000	37905	57	85	278
U4-3'Δ	0.0	51.1	9.6	683360	645455	37905	0	79	253
U4-5'ss	37.9	79.8	11.0	515875	500000	37905	73	160	289
U4-5'Δ	32.3	29.0	14.3	288602	272727	37905	112	106	376
flU4	101.1	228.0	11.0	1383359	1345454	37905	73	169	289
flU4ΔSm	0.0	174.5	17.1	1383359	1345454	37905	0	130	452
flU4-spacer	203.0	311.7	8.2	1361329	1345454	37905	149	232	217
flU4-spacer-3'ss	81.2	119.7	2.9	1361329	1345454	37905	60	89	77
flU4+D3/B	248.3	145.1	8.0	1364189	1345454	37905	182	108	210
flU4-spacer-3'ss + D3/B	120.2	112.2	2.9	1364189	1345454	37905	88	83	77

*Due to the lower MEC of 7SΔN than those of RNAs, the OD values of 7SΔN, which are at the similar level to the background, is unable to be estimated correctly compared with those of RNAs and RNA-protein complexes.

The MEC of RNA-protein is calculated by addition of the MECs of RNA and 7SΔN (or 5Sm, or 7Sm. Due to the dominancy of RNA in MEC of RNA-protein complex, using any MEC of the three protein complexes gives rise to almost identical calculated concentrations for RNA-protein complexes.)

Table S7. The calculated concentrations of RNAs and RNA-protein complexes from GFC for the competition of two RNAs' assembly into Sm subcores and cores.

the sample incubated with 7SΔN for GFC	OD(mAu) at 260nm				MEC [at 260nm]				Concentration (nM)			
	large RNA-protein	Large RNA	short RNA-protein*	short RNA*	large RNA-protein	Large RNA	short RNA-protein	short RNA	large RNA-protein	Large RNA	short RNA-protein*	short RNA*
flU4-spacer + U4-5'Δ-3'ss	122.3	141.6	26.0	29.9	1361329	1345454	288602	272727	90	105	90	110
flU4-spacer-3'ss + U4-5'Δ	78.4	123.8	30.3	27.9	1361329	1345454	288602	272727	58	92	105	102
flU4-spacer-3'ss + U4-5'Δ + D3/B	75.8	69.1	43.3	21.9	1364189	1345454	288602	272727	56	51	150	80
flU4 +U4-5'Δ + D3/B	159.2	154.3	21.6	19.9	1364189	1345454	288602	272727	117	115	75	73

*The OD values of short RNAs and short RNA-protein complexes, which are at the similar level to the background, are unable to be estimated correctly compared with those of large RNAs and large RNA-protein complexes.

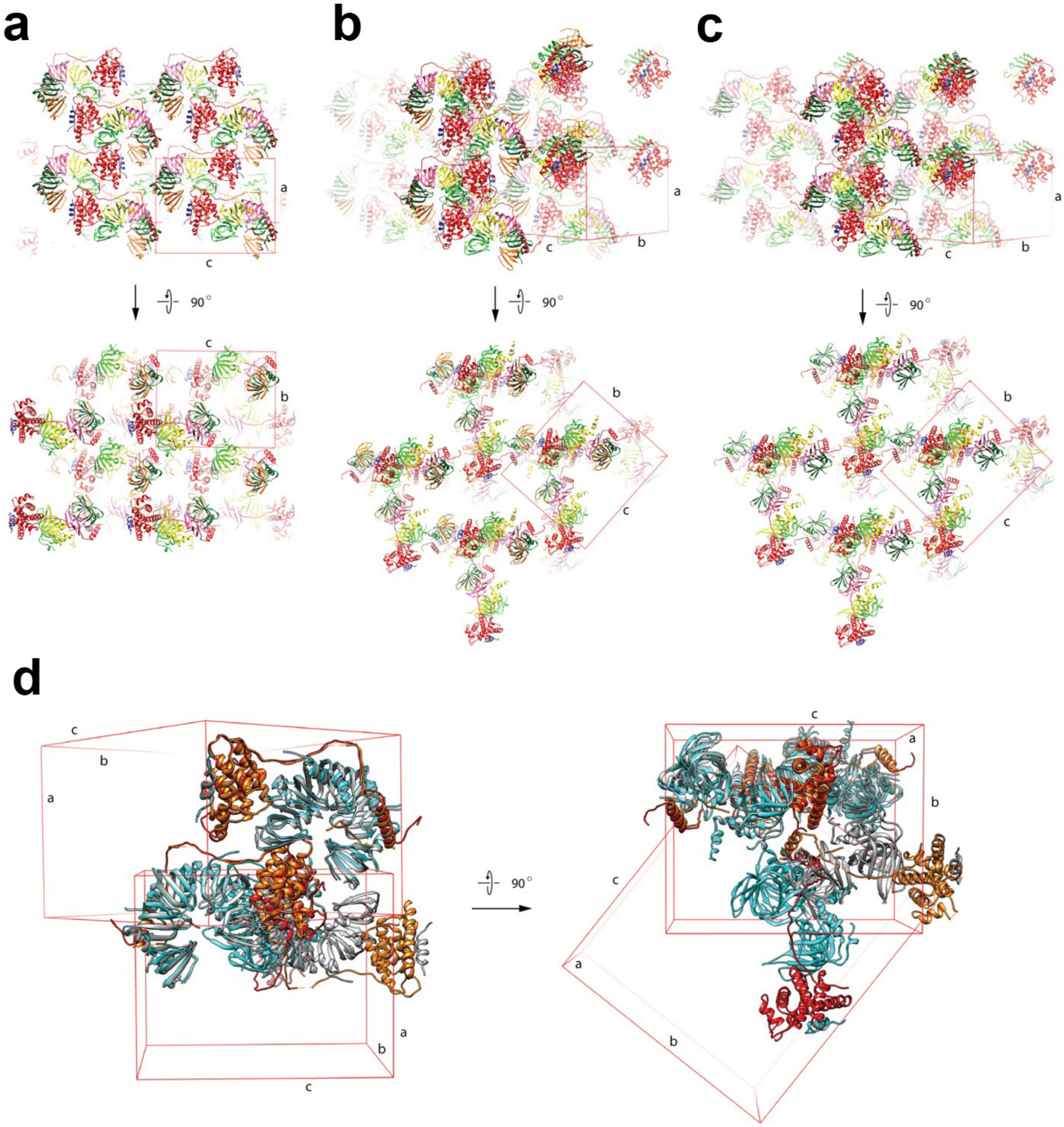

Fig. S1. Crystal packing comparison of three complexes. Two views of the crystal packing for each of 3 complexes: (a) the 7S complex from the previous study (3S6N), (b) Complex A and (c) Complex B. The five Sm proteins, D1, D2, F, E and G are colored in green, lemon, pink, dark green and orange respectively. Gemin2 and SMN_{Ge2BD} are colored in red and blue respectively. Unit cells and axis are showed. (d) Comparison of 3S6N (5Sm in light gray and Gemin2 in orange) with Complex A (5Sm in cyan and Gemin2 in red) in crystal packing. Two molecules of the complexes are superimposed in direction a.

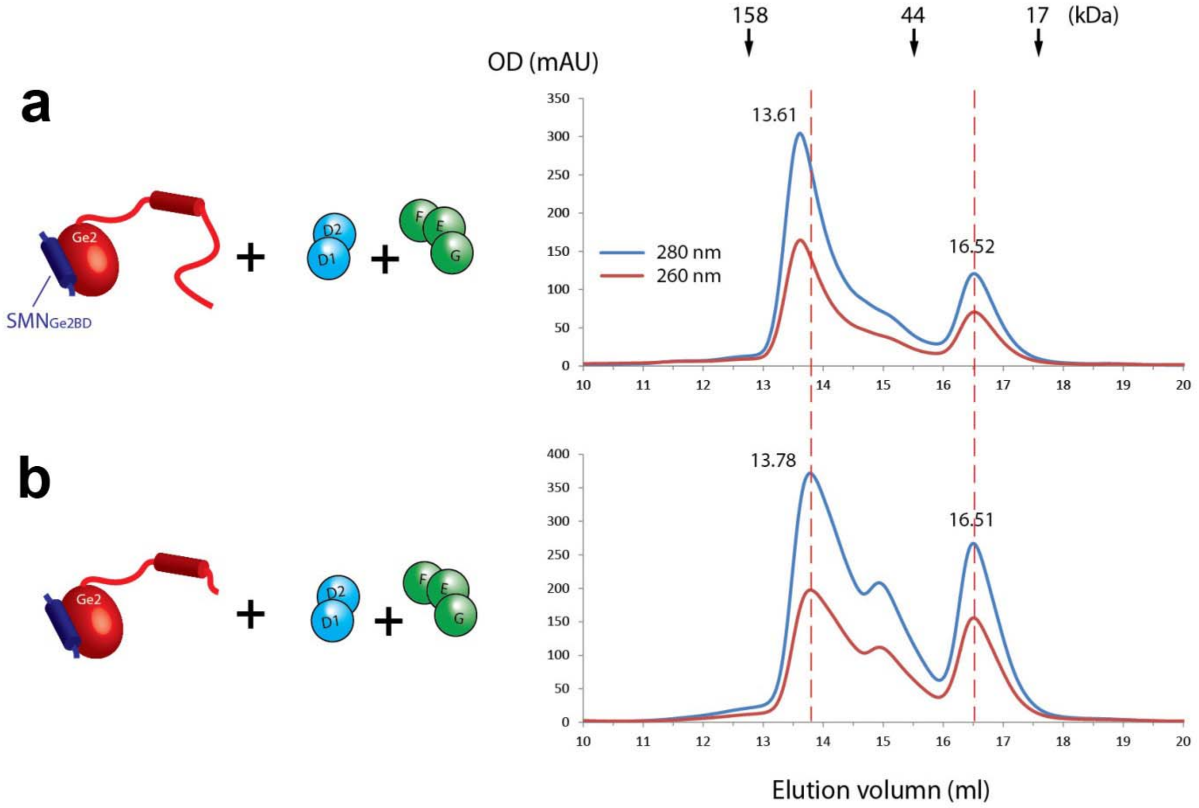

Fig. S2. The gel filtration profiles of 7S and 7SΔN. Reconstitution of 7S or 7SΔN was made by mixing extra amount of the 2 hetero-oligomeric Sm subcomplexes, D1s/D2 and F/E/G, with (a) Gemin2/SMN$_{Ge2BD}$ or (b) Gemin2ΔN39/SMN$_{Ge2BD}$ respectively followed by gel filtration chromatography separation (right panels). For each complex, one representative result from three independent experiments is shown. The input components are showed in cartoon (left panels). The positions of standard proteins are indicated at the top. Peak positions (ml) are indicated. The extra amount of D1s/D2 and F/E/G serve as inner controls (eluted at 16.5 ml). They also form small amount of D1s/D2/F/E/G heteropentamer (5Sm) at about 15.0 ml. The peaks of 7S and 7SΔN appeared at 13.61 and 13.78 ml respectively.

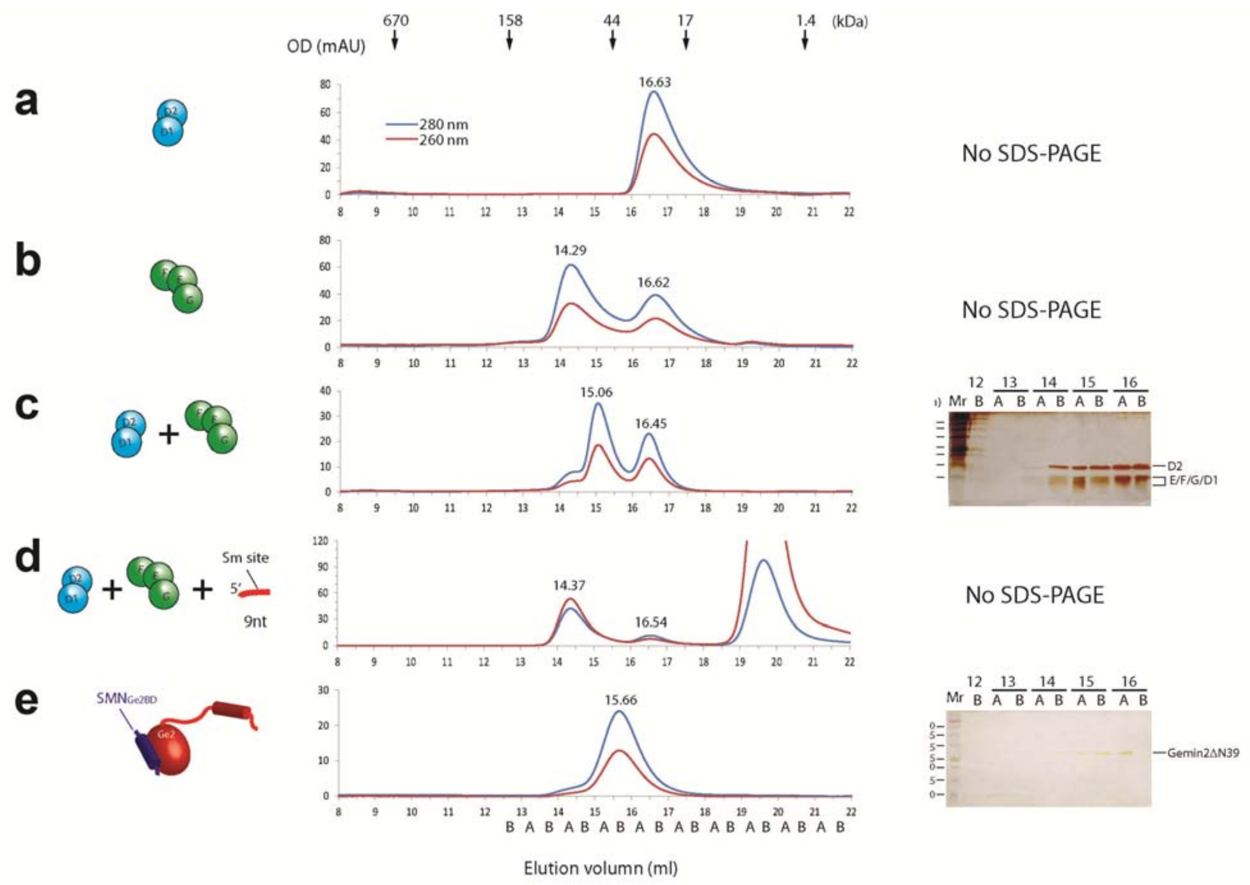

Fig. S3. The gel filtration profiles of different complexes. Different complexes (in cartoon, left panels), SmD1s/D2 (a), SmF/E/G (b), mixture of SmD1/D2 and SmF/E/G (c), mixture of 9nt and the 5 Sm proteins (d), or SMN(26-62)/Gemin2ΔN39 (e), was separated by gel filtration chromatography (middle panels) and individual fractions were analyzed by SDS-PAGE and silver staining (right panels). For each, one representative result from at least two independent experiments is shown. The positions of standard proteins are indicated at the top. Fractions (A &B) are named on the basis of volume positions (bottom). SmF/E/G (e) is the same as in Figure 2 and showed here for easy comparison. The SMN(26-62) fragment is too small to be visible on the SDS-PAGE in panel (e).

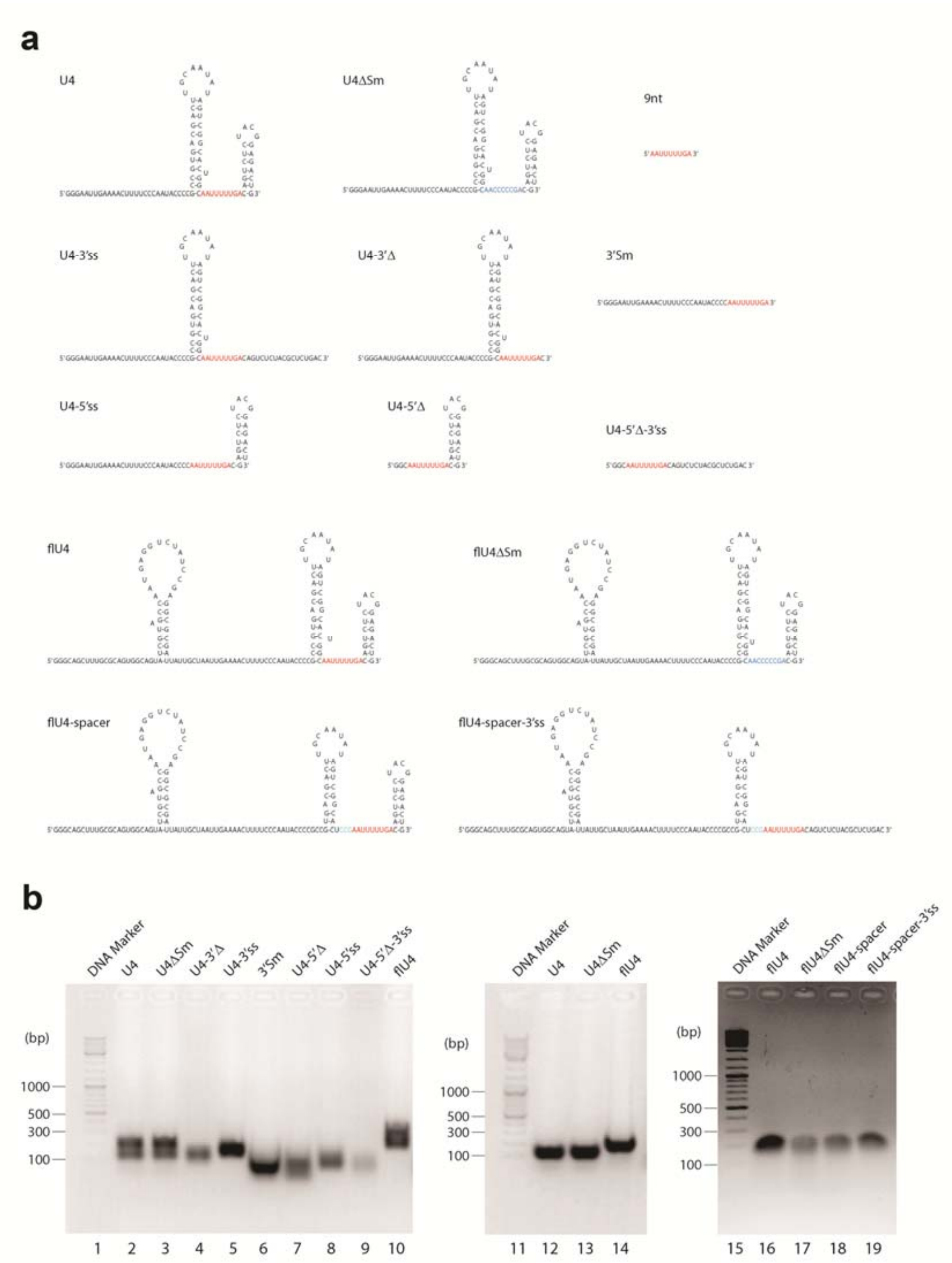

Fig. S4. The RNAs used in this study. (a) The sequence and secondary structures of the RNAs. Sm site is colored in red, ΔSm in blue, and the 3nt spacer in cyan (same as in cartoon). (b) The agarose gel electrophoresis of the *in vitro* transcribed RNAs. The RNAs in lanes 2-10 were not specially treated before loading on the gel. The three RNAs, U4, U4ΔSm and flU4, which were double-bands in lanes 2, 3 and 10, and flU4 and its three derivatives, were incubated at 65°C for 10 min and cooled to room temperature before loading on the gel (lanes 12-14 and 16-19).

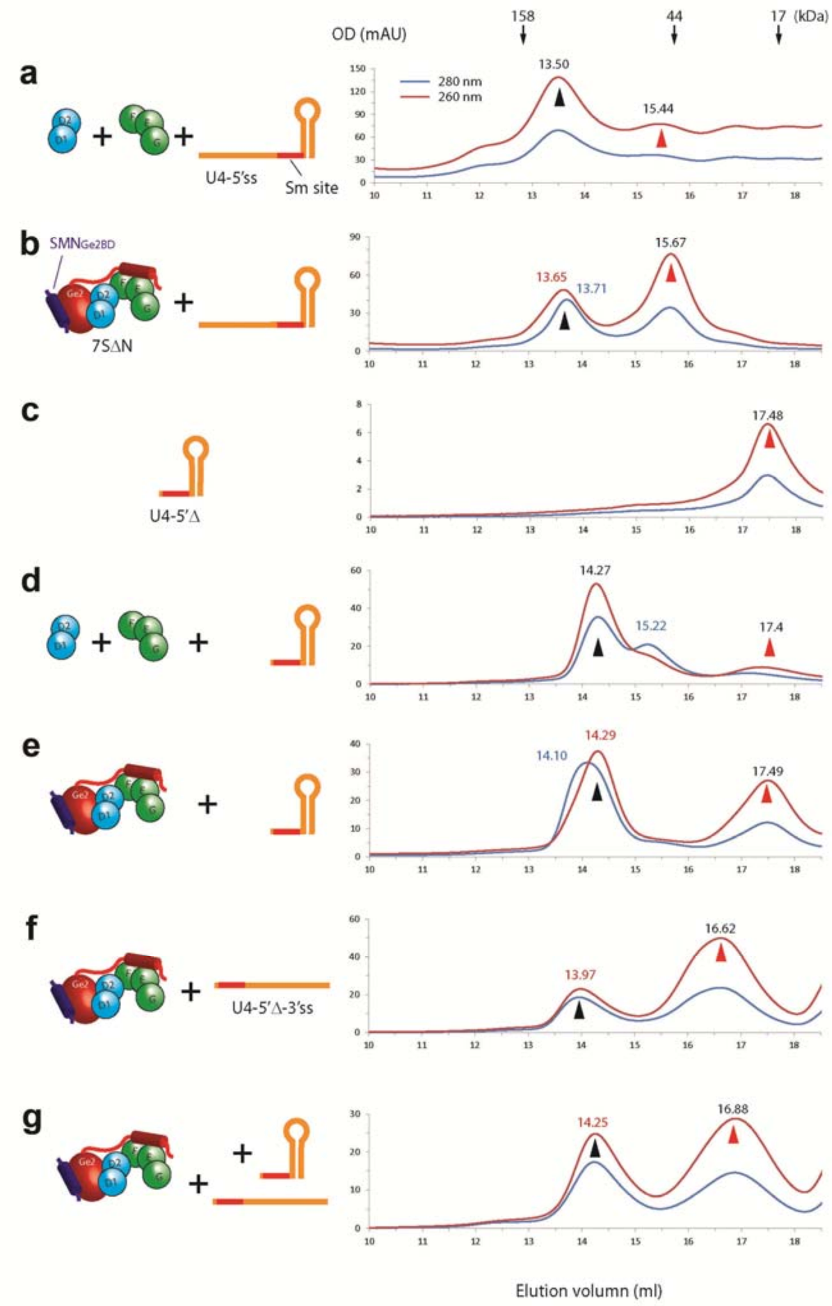

Fig. S5. The gel filtration profiles of RNA and Sm subcore formation. Mixture of 5Sm and U4-5'ss (a), mixture of 7SΔN and U4-5'ss (b), U4-5'Δ alone (c), mixture of 5Sm and U4-5'Δ (d), mixture of 7SΔN and U4-5'Δ (e), mixture of 7SΔN and U4-5'Δ-3'ss (f), or mixture of 7SΔN and equal molar amount of U4-5'Δ and U4-5'Δ-3'ss (g) was separated by gel filtration chromatography (right panels). For each, one representative result from at least two independent experiments is shown. (b) and (e) are the same as in Fig. 2 and are showed here for easy comparison. The input components are showed in cartoon (left panels). The peaks pointed at by the red arrow heads are the RNA peaks, and the peaks pointed at by the black arrow heads are the Sm-RNA complexes (subcores).

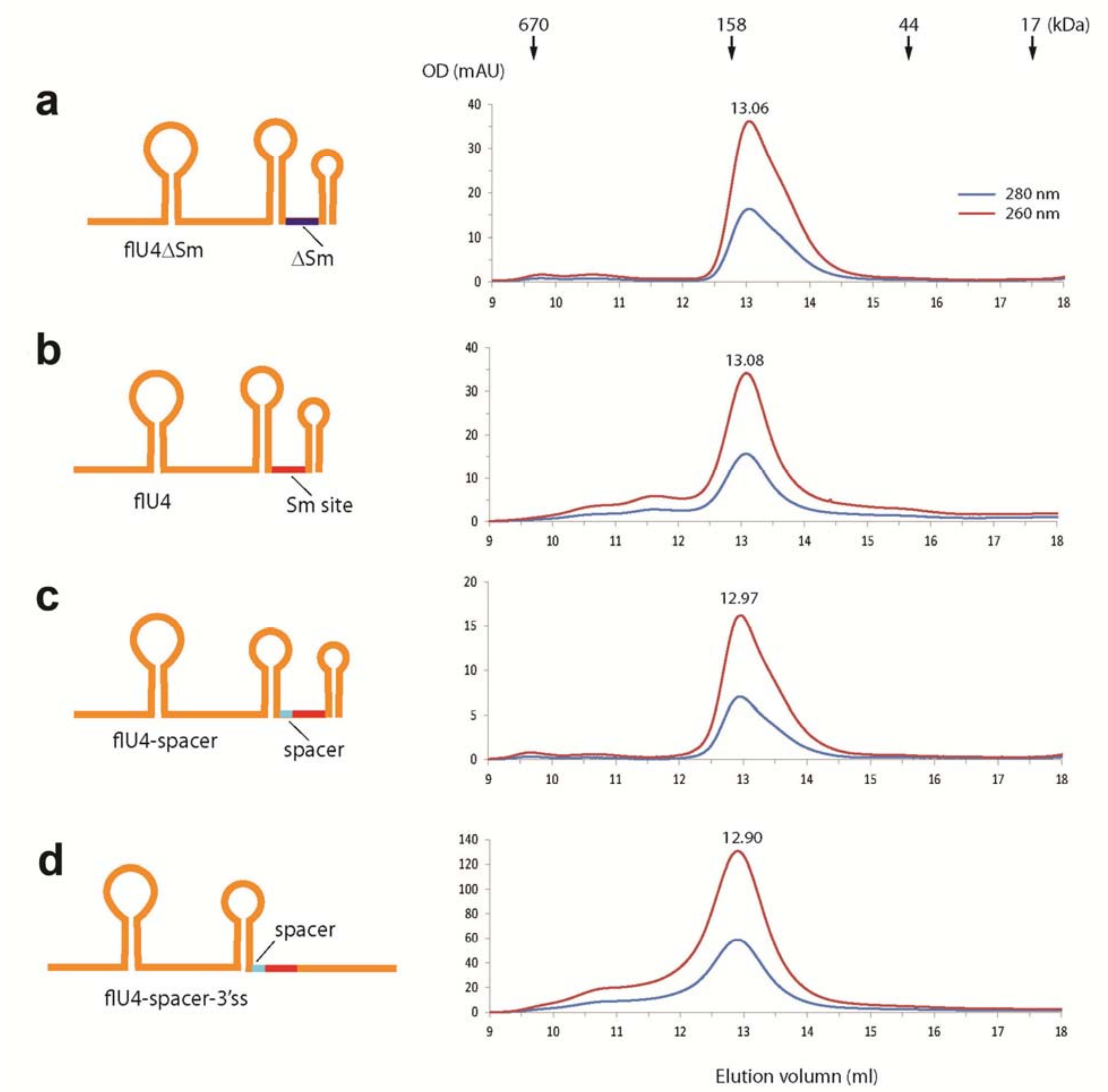

Fig. S6. The gel filtration profiles of the four large-sized RNAs. flU4ΔSm (a), flU4 (b), flU4-spacer (c) or flU4-spacer-3'ss (d) (in cartoon, left panel) was separated by gel filtration chromatography (right panels). For each, one representative result from two independent experiments is shown. The positions of standard proteins are indicated at the top.

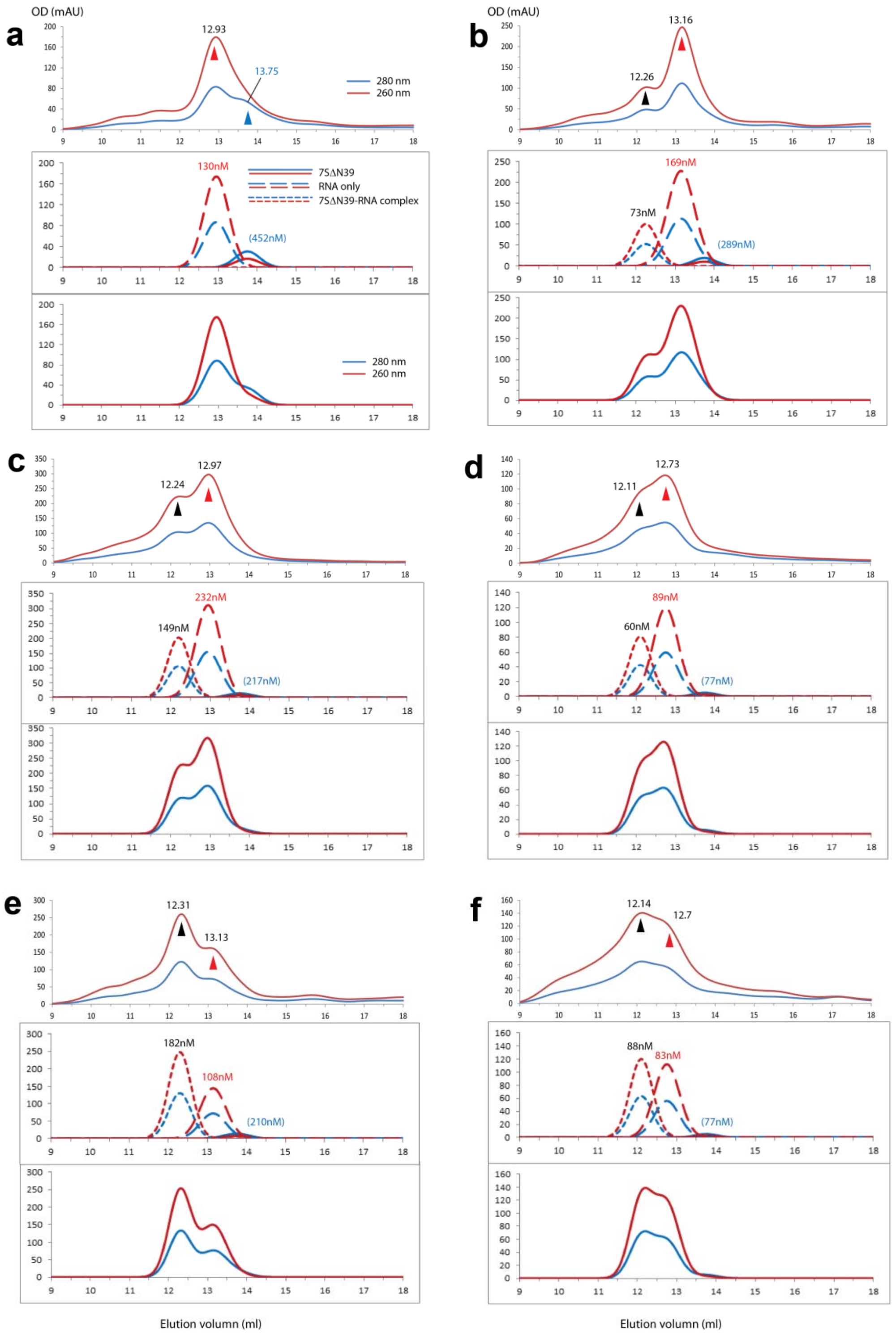

Fig. S7. The simulations of GFC traces of component peaks and the combined traces in Fig 4. Each panel corresponds to the same one in Fig.4. In each panel, the top traces are the experimental traces, the same as in Fig.4 (shown here for easy comparison), the middle traces are the simulated component peaks (and their concentrations, see Table S6 for the calculation.) and the bottom traces are the combined ones.

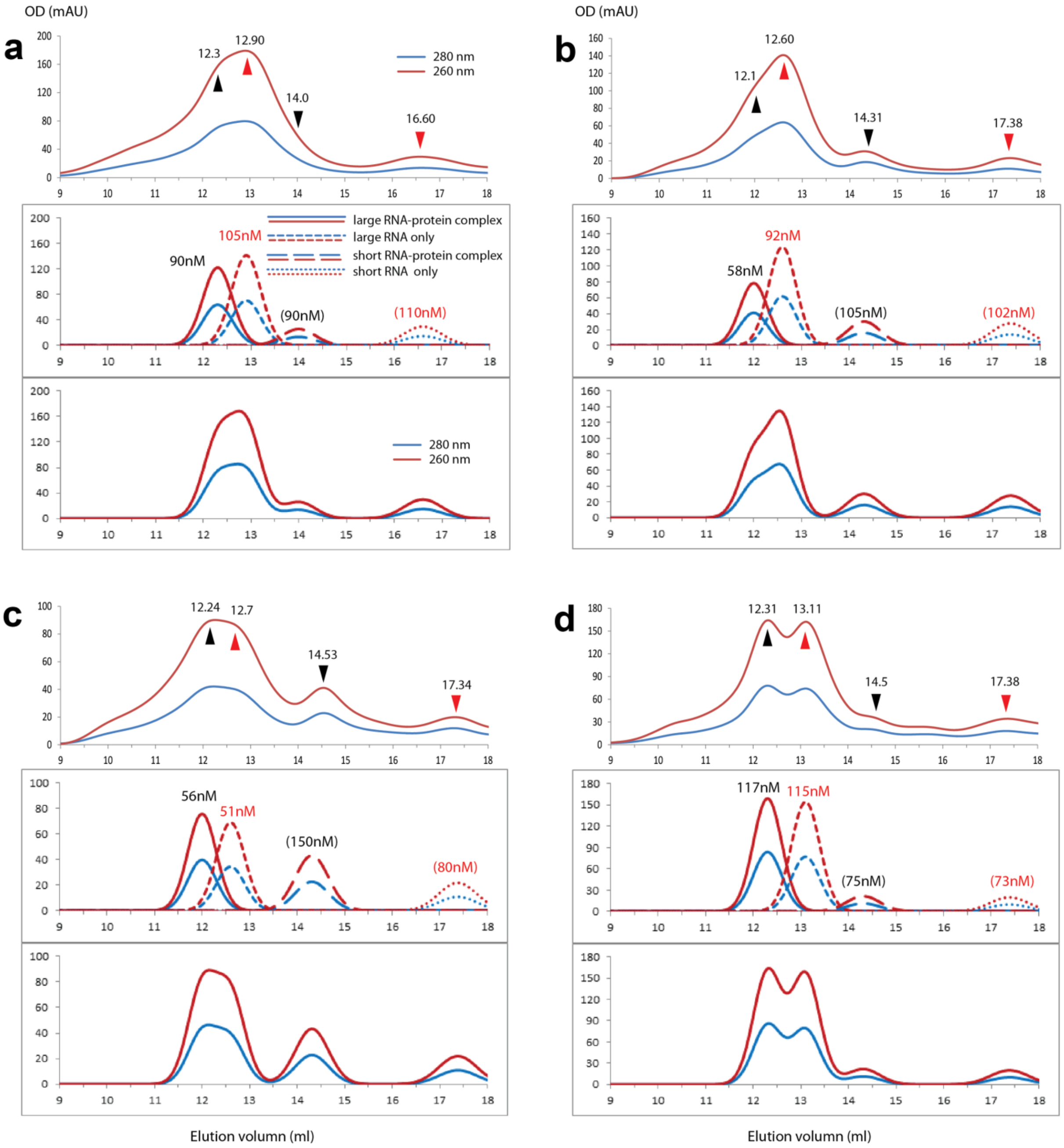

Fig. S8. The simulations of GFC traces of component peaks and the combined traces in Fig 5. Each panel corresponds to the same one in Fig.5. In each panel, the top traces are the experimental traces, the same as in Fig.4 (shown here for easy comparison), the middle traces are the simulated component peaks (and their concentrations, see Table S7 for the calculation.) and the bottom traces are the combined ones. Compared with the traces of the RNAs and RNA-protein complexes, the trace of 7SΔN is too small to contribute to the overall shape of the combined traces and is not shown in each panel.
